# Short-Axis Methyl Substitution Approach on Indacenodithiophene: A New Multi-Fused Ladder-Type Arene for Organic Solar Cells

**DOI:** 10.3389/fchem.2019.00372

**Published:** 2019-06-18

**Authors:** Yun Li, Menghan Wang, Fupeng Wu, Xuyu Gao, Sven Huettner, Youtian Tao, Zuo-Quan Jiang

**Affiliations:** ^1^Jiangsu Key Laboratory for Carbon-Based Functional Materials and Devices, Joint International Research Laboratory of Carbon-Based Functional Materials and Devices, Institute of Functional Nano and Soft Materials, Soochow University, Suzhou, China; ^2^Key Lab for Flexible Electronics, Institute of Advanced Materials, Nanjing Tech University, Nanjing, China; ^3^Macromolecular Chemistry I, Universität Bayreuth, Bayreuth, Germany

**Keywords:** indacenodithiophene, methyl group, short-axis substitution, non-fullerene electron acceptors, polymer solar cells

## Abstract

Indacenodithiophene (IDT) is a promising building block for designing organic semiconductors. In this work, a new pentacyclic ladder-type arene IDMe was designed and synthesized by introducing methyl substitution on the short-axis of IDT. Two non-fullerene electron acceptors (IDIC and ID-MeIC) without and with methyl substitution were designed and synthesized for further study. Compared with IDIC, ID-MeIC with methyl substitution on the short-axis of IDT shows smaller bandgap, stronger extinction coefficient, and better crystallinity. Besides, PBDB-T: ID-MeIC blend film shows more efficient exciton generation and dissociation and more balanced charge transport mobility. Therefore, polymer solar cells based on PBDB-T: ID-MeIC can achieve better photovoltaic performance with a PCE of 6.46% and substantial increase in *J*_SC_ to 14.13 mA cm^−2^ compared to 4.94% and 9.10 mA cm^−2^ of PBDB-T: IDIC. These results suggest that short-axis substitution on multi-fused ladder-type arenes, such as IDT is an effective way to change the optical and electronic properties of the organic semiconductors for high-performance OPVs.

**Graphical Abstract:**
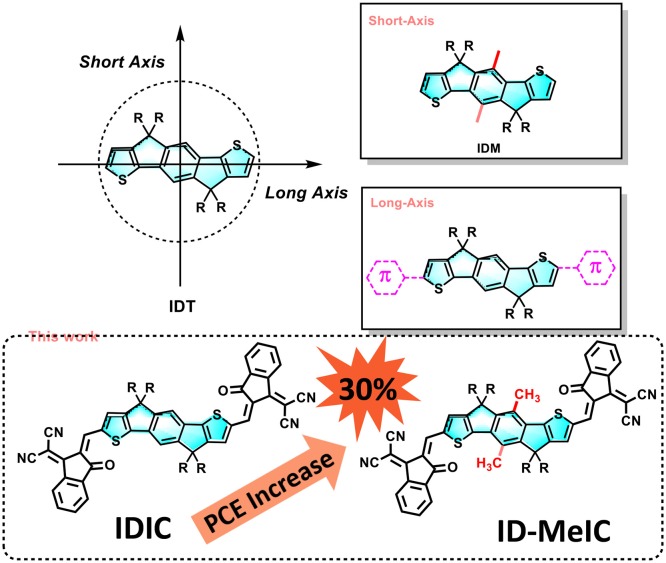


## Introduction

Organic solar cell (OSC) is one of the most promising new energy technologies due to their advantages such as low cost, lightweight, enable roll-to-roll fabrication (Hiramoto et al., 1995; Halls et al., 1996). Generally, OSCs have a bulk hetero-junction (BHC) consist of an electron donor (D) and an electron acceptor (A) (Halls et al., 1995). In the past few years, fullerene and its derivatives such as (PC_61_BM) are widely applied as the electron acceptor (Chen et al., 2015). Although the PCE of fullerene-based OSCs can above 11% (Zhao et al., 2016), it is hard to achieve a further development because of its complicated synthesis, low light absorption coefficient, and poor tunability in energy levels (He et al., 2010). In the past 3 years, non-fullerene electron acceptors (NFAs) as alternatives for fullerene-base electron acceptors have achieved great developments. Now, the maximum PCEs have surpassed 15% (Yuan et al., 2019) for single-junction cells and 17% for double-junction tandem cells (Meng et al., 2018). Among all kinds of NFAs, A-D-A type NFAs is one of the most successful design strategies (Kang et al., 2016; Li S. et al., 2016; Li Z. et al., 2016; Li et al., 2018; Xie et al., 2018a,b). What is particularly exciting is the chemistry that has allowed the synthesis of novel multi-fused ladder-type arenes with tunable energy levels and highly desirable optical or electronic properties for A-D-A NFAs (Lin et al., 2015a; Wu et al., 2015; Dai et al., 2017; Yang et al., 2018).

Indacenodithiophene (IDT), a pentacyclic ladder-type arene (Bai et al., 2015; Lin et al., 2015b), has a coplanar fused-ring aromatic structure with a low degree of energetic disorder and high carrier mobility (Würfel et al., 2015; Jia et al., 2017). Thus, IDT has been widely applied as a donor (D) unit and combined with an acceptor (A) unit to construct a variety of D-A conjugated polymers, which become one of the major families of polymers for organic solar cells (OSCs) (Li et al., 2017a; Zhang et al., 2017). In these 2 years, various non-fullerene electron acceptors based on IDT- and its derivatives have also been developed for OSCs (Yan et al., 2017; Jiang et al., 2018). These facts are enough to prove that IDT is a promising building block to design organic semiconductors for organic electronics. Thus, it is valuable to tailor the IDT's structure for selectively optimizing electrical properties of IDT-based organic semiconductors.

**Figure 1:**
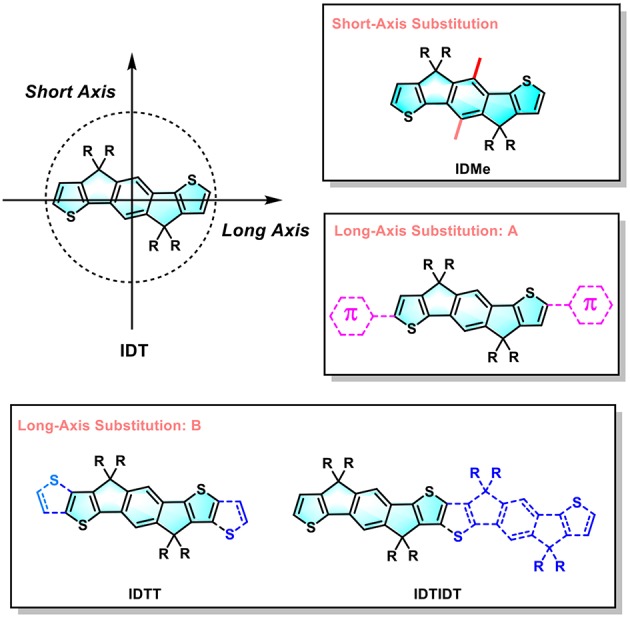
Design strategy of IDT-derivatives.

In the past 3 years, the structural diversity of IDT is flourishing (Liu et al., 2017). However, as shown in Figure 1, most of IDT-derivatives focus on the long-axis substitution approach such as through extension conjugated length along the long axis (Li et al., 2017b; Wang et al., 2017). Many researchers may notice that there are possible substituted positions on the central benzene of IDT core (Li et al., 2019). However, few related reports are available in this short-axis substitution approach probably due to synthesis difficulty. Dithienyl[1,2-b:4,5-b′] benzodithiophene (BDCPDT), another kind of core, can easily make this modification by using different side-chains in the precursors (Kan et al., 2018). Recently, our group designed and synthesized two new non-fullerene electron acceptors (CBT-IC and SBT-IC) by the short-axis substitution modification of non-fullerene acceptors based on the BDCPDT-system. This work shows that the short-axis substitution is also an effective way to optimize the optical and electronic properties of the organic semiconductors for organic photovoltaics (OPVs) (Lin et al., 2018). Besides, unlike the side-chains on the sp^3^ hybridized carbon that make them point out of the backbone planes; short-axis substitution can provide another kind of side-chains appended to the conjugated backbone; thus, they can directly affect energy levels especially the HOMO (Yang et al., 2016; Bin et al., 2017). This impact is quite similar to that of end-capping groups which are placed along the IDT's long-axis.

Herein, a new IDT-derivative core, denoted as IDMe, was design and synthesized through the short-axis methyl substitution approach (introducing the methyl on the central benzene of IDT core, Figure 1). Then, a new non-fullerene acceptor (ID-MeIC) based on IDMe was design and synthesized. For comparison, IDIC without methyl-substitution was also synthesized. Compared with IDIC, ID-MeIC with methyl substitution on short-axis of IDT shows a smaller bandgap, stronger extinction coefficient and better crystallinity. Besides, PBDB-T: ID-MeIC blend film shows more efficient exciton generation and dissociation a balanced charge transport mobility. Therefore, polymer solar cells based on PBDB-T: ID-MeIC can achieve better photovoltaic performance with a PCE of 6.46%, a substantial increase in *J*_SC_ (14.13 mA cm^−2^) and a higher *V*_OC_ of 0.90 V while a smaller *FF* of 0.50. The value of PCE is 30% higher than that of PBDB-T: IDIC based PSCs.

## Results and Discussion

### Material Synthesis and Characterization

**Figure 2:**
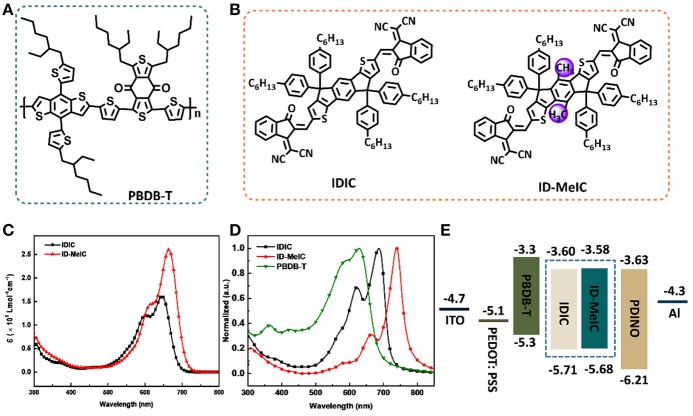
Chemical structures of **(A)** donor PBDB-T and **(B)** acceptor IDIC and ID-MeIC; **(C)** The molar extinction coefficients of IDIC and ID-MeIC in chloroform (10^−5^ M); **(D)** Normalized absorption spectra of IDIC and ID-MeIC in thin film; **(E)** Energy diagram of materials used in the PSC.

**Scheme 1:**
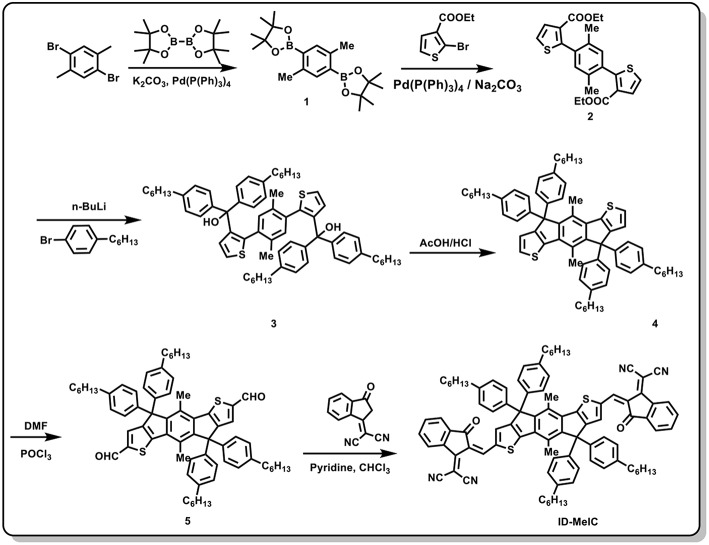
Synthetic routes of ID-MeIC.

Chemical structures of polymer donor (PBDB-T) and two non-fullerene electron acceptors (IDIC and ID-MeIC) are shown in Figure 2. The detail synthetic routes of compound ID-MeIC are illustrated in Scheme 1. The key intermediate compound 2 was obtained via Suzuki coupling reaction with 92% yield. Then 4-hexylphenyl was introduced into compound 2 via nucleophilic reaction, followed by intramolecular Friedel-Crafts cyclization under acidic conditions to afford the new building block (IDMe) with 68% yield in two steps. Compound 5 (IDMe-CHO) was obtained by Vilsmeier-Haack reaction using dimethylformamide (DMF) and phosphorus oxychloride (POCl_3_) with 80% yield. Finally, ID-MeIC was synthesized by the Knoevenagel reaction of IDMe-CHO and 1,1-Dicyanomethylene-3-indanone (IC) in chloroform with 80% yield. For comparison, IDIC was also synthesized according to the literature report (Lin et al., 2017). All the chemically synthesized compounds were fully characterized by ^1^H NMR and MALDI-TOF with their spectra described in the Supporting Information. Thermogravimetric analysis (TGA) was performed to study the thermal stability of ID-MeIC. As shown in Figure S1, ID-MeIC shows decomposition temperature (*T*_d_, 5% weight-loss) of 353°C, which indicated that ID-MeIC has good thermal stability.

As shown in Figure 2C, with the detail spectra data provided in Table 1. IDIC show absorption band in 500–700 nm with a maximum absorption wavelength at 646 nm. However, ID-MeIC has the maximum absorption wavelength red-shifting to 665 nm with an extinction coefficient of 2.61 × 10^5^ L mol^−1^ cm^−1^, which is larger than that of IDIC (1.63 × 10^5^ L mol^−1^ cm^−1^). In thin film, the maximum absorption wavelength of IDIC and ID-MeIC red-shift to 689 nm, 741 nm, respectively (Figure 2D). Interestingly, ID-MeIC shows larger bathochromic (about 76 nm) and stronger relative intensity of absorption peak in the long wavelength. This may due to the stronger intermolecular aggregation interaction by introducing the methyl substitution on the short-axis of IDT. The optical band gaps ($E_{\text{g}}^{\text{opt}}$) of IDIC and ID-MeIC calculated from their absorption edge are 1.67 and 1.51 eV, respectively. Meanwhile, the energy-level alignment of IDIC and ID-MeIC are calculated by the cyclic voltammetry measurements and the potentials are calibrated using Fc/Fc^+^ redox couple (Figure S2). The corresponding highest occupied molecular orbital (HOMO) and lowest unoccupied molecular orbital (LUMO) energy levels are calculated from the onset oxidation and reduction potentials (ϕ_ox/red_), according to the equations: *E*_HOMO/LUMO_ = -e (ϕ_ox/red_ + 4.8–$\phi_{\text{Fc}/\text{Fc}}^{+}$) (eV). The *E*_HOMO/LUMO_ are calculated to be −5.68/−3.58 eV and −5.71/−3.60 eV for IDIC and ID-MeIC, respectively. The higher *E*_HOMO_ and deeper *E*_LUMO_ of ID-MeIC indicate that methyl substitution on the short-axis of IDT can directly affect the energy levels.

**Table 1:** Optical and electrochemical properties of IDIC and ID-MeIC.

**Acceptor**	***λ*_max_^sol(a)^**	***λ*_max_^flim(b)^**	***λ*_onset_^flim(c)^**	**E_*g*_^opt(d)^**	***E*_**HOMO (e)**_**	***E*_**LUMO (e)**_**	**E_*g*_^cv(f)^**
	**(nm)**	**(nm)**	**(nm)**	**(eV)**	**(eV)**	**(eV)**	**(eV)**
IDIC	646	689	741	1.67	−5.71	−3.60	2.11
ID-MeIC	665	741	819	1.51	−5.68	−3.58	2.10

(a) Maximum absorption in chloroform solution;

(b) Maximum absorption at thin film;

(c) Absorption onset at thin film;

(d) Optical band gap calculated by the absorption onset of thin film state;

(e) HOMO/LUMO value calculated from the onset oxidation/reduction potential;

(f) *HOMO-LUMO gap estimated from CV*.

To investigate the geometric structure and electronic properties of IDIC and ID-MeIC, density functional theory (DFT) calculations at the B3LYP/6-31G (d) level were performed. As shown in Figure S3, IDIC and ID-MeIC both have highly coplanar structure. And the HOMO/LUMO wave functions of compound IDIC and ID-MeIC are well delocalized over their backbone, which is beneficial for their charge transport mobilities. In addition, the calculated HOMO/LUMO of IDIC and ID-MeIC are −5.63/−3.39 eV and −5.54/−3.34 eV, respectively, which are consistent with values estimated from CV experiments. These results suggest that methyl substitution on short-axis of IDT methyl can increase the electron-donating ability of IDT core and directly affect its energy levels.

**Figure 3:**
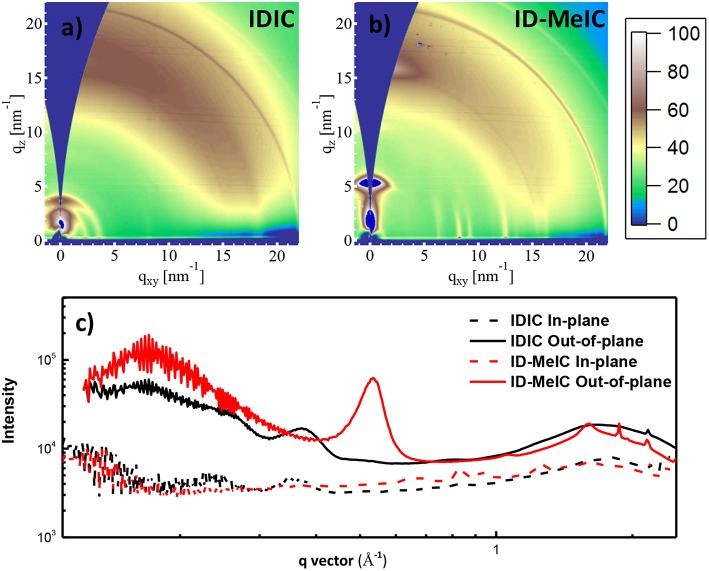
2D GIWAXS patterns of **(a)** IDIC film and **(b)** ID-MeIC film; **(c)** Sector average line outs of the GIWAXS images of IDIC and ID-MeIC neat film.

The methyl group has a tremendous impact on the crystallinity and molecular packing in the thin films, which be evidenced by grazing-incidence wide-angle X-ray scattering (GIWAXS) as shown in Figures 3a–c. For IDIC, both the lamellar (100) reflections and π-π stacking (010) reflections can be observed, which suggested that face-on and edge-on crystallites coexisted in the corresponding film. However, ID-MeIC with methyl substituents on the central benzene ring along the short-axis of IDT, displayed a predominant face-on crystalline orientation. Interestingly, ID-MeIC exhibited stronger π-π stacking (010) reflection with a new strong reflection peak appear in the out-of-plane direction. These results suggest methyl substituents on the short-axis can enhance the crystallinity of IDT.

### Photovoltaic Performances

To investigate the photovoltaic performance of these NF-SMAs, polymer solar cells were fabricated with the device structure of indium tin oxide(ITO)/poly(3,4-ethylene dioxythiophene): poly(styrene sulfonate)(PEDOT: PSS)/Active Layer/3,3′-(1,3,8,10-Tetraoxoanthra[2,1,9-def: 6,5,10-d'e'f']diisoquinoline-2,9(1H,3H,8H,10H)-iyl)bis(N,N-dimethylpropan-1-amine oxide)(PDINO)/aluminum (Al), where PEDOT:PSS and PDINO served as the anode and cathode interlayers; a wide bandgap polymer donor named PBDB-T blending with these two NF-SMAs served as the active layer. The devices were optimized with different donor/acceptor (D/A) weight ratios, thermal annealing conditions, and active layer thickness. The detailed of optimization process are shown in ESI^†^ Tables S1–S4.

**Figure 4:**
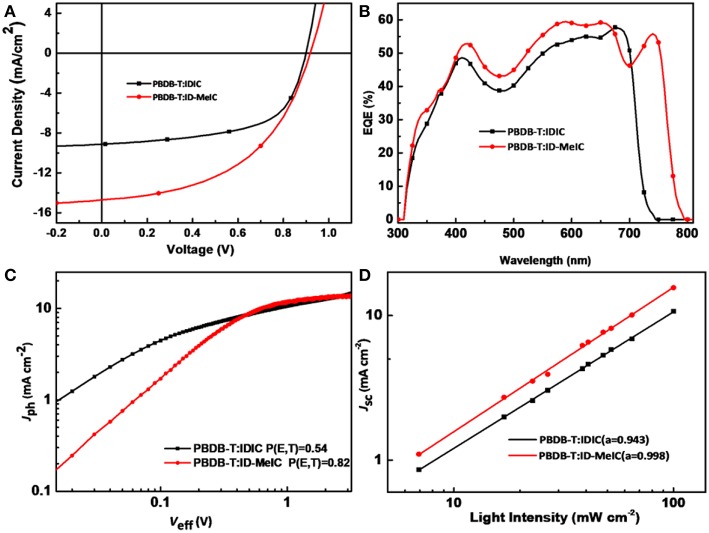
IDIC and ID-MeIC-based devices **(A)** *J–V* curves; **(B)** EQE curves; **(C)** Photocurrent vs. effective voltage; **(D)** Light-intensity dependence of *J*_SC_.

The current density-voltage (*J-V*) curves are shown in Figure 4A with the detail photovoltaic performance parameters provided in Table 2. For IDIC without methyl substitution, the optimized PBDB-T: IDIC -based device shows a maximum PCE of 4.94% with a *V*_OC_ of 0.89 V, a *J*_SC_ of 9.10 mA cm^−2^ and an *FF* of 0.61. In contrast, for ID-MeIC, the PBDB-T: ID-MeIC -based device show the highest PCE of 6.46% with a substantial increase in *J*_SC_ (14.13 mA cm^−2^) and a slightly higher *V*_OC_ of 0.90 V whereas a decrease of *FF* (0.50). This higher *J*_SC_ and *V*_OC_ of PBDB-T: IDIC-Me -based device is due to the methyl substitution on the short-axis of IDT which can elevate the *E*_HOMO_ and decrease the *E*_g_ of ID-MeIC acceptor. Figure 4B depicts the external quantum efficiency (EQE) curve of this device. It is obvious that device based on PBDB-T: ID-MeIC demonstrates has a strong solar spectral response at the range of 700–800 nm, which is in consistency with the UV-vis spectra and is accounted for its higher *J*_SC_. The integrated current density *J*_SC_ of PBDB-T: IDIC and PBDB-T: ID-MeIC-based devices are calculated of 10.47, 13.03, mA cm^−2^, respectively.

**Table 2:** Comparison photovoltaic parameters of PSCs based on IDIC and ID-MeIC.

**Active layer**	***V*_**OC**_ (V)**	***J*_SC_(mA cm^**−2**^)**	***J*_SC_ (mA cm^**−2**^) ^**(a)**^**	***FF***	**PCE_**max**_/PCE ^**(b)**^_**avr**_ (%)**
PBDB-T: IDIC	0.89 ± 0.00 (0.89)	8.67 ± 0.43 (9.10)	10.47	0.60 ± 0.01 (0.61)	4.94/4.67
PBDB-T: ID-MeIC	0.90 ± 0.00 (0.90)	13.67 ± 0.46 (14.13)	13.03	0.50 ± 0.01 (0.50)	6.46/6.28

(a) Integrated current density calculated from EQE curves;

(b) *Average values were obtained from 10 devices*.

### Charge Generation, Transport, and Recombination

The photocurrent density (*J*_ph_) vs. effective voltage (*V*_eff_) of the devices based on PBDB-T: acceptors were also plotted to study the exciton dissociation and charge collection properties in the OSCs (Figure 4C). *J*_ph_ were measured as a function of the effective voltage (*V*_eff_ is defined as *V*_0_-*V*_bias_), from which we can calculate the exciton dissociation probability (P_diss_ defines as the ratio of *J*_ph_/*J*_sat_, and *J*_sat_ is the saturation current density at high *V*_eff_). In this way, under short-circuit conditions, the values of P_diss_ were determined to be 54% and 82% for the PBDB-T: IDIC and PBDB-T: ID-MeIC–based devices, respectively. These results indicate that PBDB-T: ID-MeIC–based device has more efficient exciton generation and dissociation, which is contributed to its high *J*_SC_.

To study the charge recombination kinetics properties in the devices we measured the dependence of *J*_SC_ on light intensity (P_light_); There the power-law equation *J*_SC_ ∝ P^α^ can be described the relationship between *J*_SC_ and P_light_. As we know, if the value of α is close to 1, it means that all the charges are swept out and collected by the electrode without recombination in the device. As shown in Figure 4D, the value of α for PBDB-T: IDIC and PBDB-T: ID-MeIC–based devices are 0.943 and 0.998, respectively. These results imply that methyl substitution on the short-axis of IDT help prevent the bimolecular recombination.

The charge transport properties of IDIC and ID-MeIC were measured by space charge limited current (SCLC) measurements (Figure S4). The electron mobility (μ_e_) of IDIC and ID-MeIC are 6.6 × 10^−6^ and 1.0 × 10^−6^ cm^2^ V^−1^ s^−1^, respectively. The hole (μ_h_) and electron mobilities of the blend films also measured to investigate the charge-transport behavior in the active layer. As shown in Figure S4, the μ_h_/ μ_e_ of PBDB-T: IDIC and PBDB-T: ID-MeIC–based devices were evaluated to be 2.1 × 10^−4^/7.8 × 10^−6^ and 9.1 × 10^−5^/3.4 × 10^−6^ cm^2^V^−1^S^−1^ with a μ_h_/μ_e_ ratio of 27 and 26, respectively. The slightly balanced charge transport proprieties of PBDB-T: ID-MeIC-based devices also contribute to its high *J*_SC_.

### Morphology Characterization

**Figure 5:**
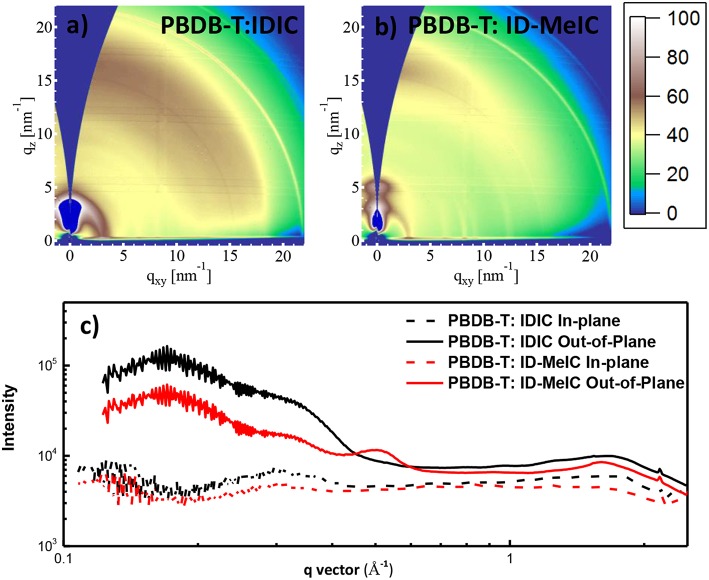
2D GIWAXS patterns of **(a)** PBDB-T: IDIC, **(b)** PBDB-T: ID-MeIC blend film; **(c)** Sector average line outs of the GIWAXS images of PBDB-T: IDIC and PBDB-T: ID-MeIC blend film.

The morphologies of PBDB-T: IDIC and PBDB-T: ID-MeIC blend films were firstly investigated by GIWAXS (Figure 5). Similarly, both the lamellar (100) reflections and π-π stacking (010) reflections were competitively observed for PBDB-T: IDIC blend film, confirming that face-on and edge-on crystallites coexisted. From Figure 5b, it is obvious that PBDB-T: ID-MeIC blend film has a predominant face-on crystalline orientation. This face-on crystalline orientation is beneficial for the charge transport in the solar cells. On the other hand, for PBDB-T: ID-MeIC blend film, polymer PBDB-T shifts too much lower q in the (010) direction corresponding to larger π-π distance and a smaller coherence length (CL) than that of PBDB-T: IDIC and PBDB-T: IDIC-Me blend film. These results indicate that PBDB-T has not good compact molecular packing when blending with ID-Me IC, which also accounts for the lower FF based on PBDB-T: ID-MeIC–based device.

**Figure 6:**
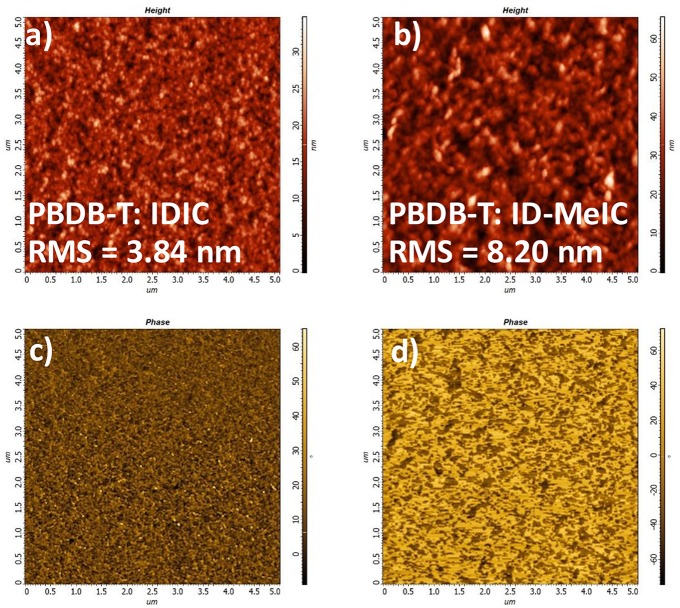
AFM height images and phase images of **(a,c)** PBDB-T: IDIC, **(b,d)** PBDB-T: ID-MeIC.

The morphologies of the blend films PBDB-T: IDIC and PBDB-T: ID-MeIC were also investigated by atomic force microscopy (AFM). As shown in Figure 6. The excessive crystalline feature of ID-MeIC in the thin film is revealed by its larger root-mean-square (RMS) roughness of 8.20 nm over that of the IDIC film (3.84 nm). This larger RMS roughness of ID-MeIC is another factor that causes the low FF of the device based on PBTB-T: ID-MeIC blend films.

## Conclusion

In summary, a new pentacyclic ladder-type arene ID-MeIC has been design and synthesized by methyl substitution on the short-axis of IDT core. ID-MeIC was functionalized as non-fullerene electron acceptors for comparison with previously reported IDIC without the methyl substitution. Compared with IDIC, ID-MeIC with methyl substitutionon on short-axis of IDT showed smaller bandgap, stronger extinction coefficient, better crystallinity. Besides, PBDB-T: ID-MeIC blended film exhibited more efficient exciton generation and dissociation as well as more balanced charge transport ability. Therefore, polymer solar cells based on PBDB-T: ID-MeIC demonstrated better photovoltaic performance with higher PCE of 6.46% and a substantial increase of *J*_SC_ to 14.13 mA cm^−2^. The value of PCE is 30% higher than that of the PBDB-T: IDIC based PSC. Our results suggested that IDMe is a promising multi-fused ladder-type building block for designing efficient non-fullerene electron acceptors. And short-axis substitution on multi-fused ladder-type arenes, such as IDT and IDTT is an effective way to tune the optical and electronic properties of the organic semiconductors for high-performance OPVs.

## Data Availability

The raw data supporting the conclusions of this manuscript will be made available by the authors, without undue reservation, to any qualified researcher.

## Author Contributions

YL: preparation and characterization of materials. MW: device preparation and characterization. FW: synthesis of materials. XG: morphology characterization. SH: morphology characterization. YT: guide device preparation. Z-QJ: guide material synthesis.

### Conflict of Interest Statement

The authors declare that the research was conducted in the absence of any commercial or financial relationships that could be construed as a potential conflict of interest.
