# Supplementary material for: Short-Axis Methyl Substitution Approach on Indacenodithiophene: A New Multi-Fused Ladder-Type Arene for Organic Solar Cells
Source: Front Chem. 2019 Jun 18;7:372. doi: 10.3389/fchem.2019.00372 (PMC6611393; doi:10.3389/fchem.2019.00372)
Supplement: Supplementary file 1 [file Table_1.DOCX]

**Short-Axis** **Methyl Substitution Approach on Indacenodithiophene: A New Multi-Fused Ladder-Type Arene for Organic Solar Cells**

**Yun Li,^1^ Menghan Wang,^2^ Fupeng Wu,^1^ Xuyu Gao,^2^ Sven Huettner,^3^ Youtian Tao,^*2^ Zuo-Quan Jiang^*1^**

^1^Institute of Functional Nano & Soft Materials (FUNSOM), Jiangsu Key Laboratory for Carbon-Based Functional Materials & Devices, Joint International Research Laboratory of Carbon-Based Functional Materials and Devices, Soochow University, 199 Ren'ai Road, Suzhou, 215123, Jiangsu, P.R. China.

*E-mail:* [*zqjiang@suda.edu.cn*](mailto:zqjiang@suda.edu.cn)

^2^Key Lab for Flexible Electronics & Institute of Advanced Materials, Nanjing Tech University, 30 South Puzhu Road, Nanjing, 211816, P. R. China.
 *E-mail:* [*iamyttao@njtech.edu.cn*](mailto:iamyttao@njtech.edu.cn)

^3^Macromolecular Chemistry I, Universität Bayreuth, 30 Universitätsstr, 95447 Bayreuth, Germany

**Keywords: Indacenodithiophene, Short-axis substitution, Non-fullerene electron acceptors, Polymer solar cells**

**Materials and Characterization:**

All starting materials and reagents were purchased from commercial sources and used without further purification unless otherwise specified. Ethyl 2-bromothiophene-3-carboxylate were synthesized according to previously reported methods.

Scheme S1. The synthetic routes for ID-MeIC.

**Synthesis of Compound 1.**

The 1,4-dibromo-2,5-dimethylbenzene (12.50 g, 47.35 mmol) was dissolved in dioxane and the 4,4,4',4',5,5,5',5'-octamethyl-2,2'-bi(1,3,2-dioxaborolane) (30.10 g, 118.42 mmol), was then added. The mixture was deoxygenated by bubbling nitrogen gas through it for 30 minutes and then Pd(PPh_3_)_4_ (1.18 g, 0.94 mmol) and K_2_CO_3_ (6.53 g, 47.35 mmol) were added. the resulted mixture was heated at 120 °C overnight. After cooling to room temperature, the resulted mixture was extracted with DCM. The organic phase was collected and dried over anhydrous Na_2_SO_4_. Then, the solvent was evaporated, and the crude product was purified on a silica gel chromatography using 2:3(DCM: PE) as the eluent. Evaporation of the solvent yielded the product as a white solid (15.26 g, 90%). ^1^H NMR (600 MHz, CDCl_3,_ δ): 7.53 (s, 2H), 2.47 (s, 6H), 1.33 (s, 24H).

**Synthesis of Compound 2.**

The compound 1 (5.00 g, 13.96 mmol) was dissolved in toluene (10 mL)/H_2_O (5 mL)/EtOH (5 mL) and the ethyl 2-bromothiophene-3-carboxylate (8.16 g, 34.89 mmol) was then added. The mixture was deoxygenated by bubbling nitrogen gas through it for 10 minutes and then Pd(PPh_3_)_4_ (0.26 g, 0.28 mmol) and Na_2_CO_3_ (7.39 g, 69.80 mmol) were added. The resulted mixture was heated at 100 °C for 8 h. After cooling to room temperature, the resulted mixture was extracted with DCM. The organic phase was collected and dried over anhydrous Na_2_SO_4_. Then, the solvent was evaporated, and the crude product was purified on a silica gel chromatography using 2:3 (DCM: PE) as the eluent. Evaporation of the solvent yielded the product as a white solid (5.32 g, 92%). ^1^H NMR (600 MHz, CDCl_3_, δ): 7.54 (d, *J* = 6 Hz, 2H), 7.27 (d, *J* = 6 Hz, 2H), 7.13 (s, 2H), 4.15 (m, 4H), 2.14 (s, 6H), 1.13 (t, *J* = 6 Hz, 6H).

**Synthesis of Compound 4.**

1-bromo-4-hexylbenzene (13.90 g, 57.96 mmol) was dissolved in anhydrous THF (60 mL) and placed under nitrogen atmosphere. The solution was cooled to -78 °C and stirred while 2.4 M n-butyllithium in hexane (24.00 mL, 57.96 mmol) was added dropwise. The mixture was stirred for 1 h at -78 °C, and then a solution of compound 2 (4.00 g, 9.66 mmol) in THF (40 mL) was added dropwise. The reaction was warmed to room temperature and stirred overnight, and then poured into water and extracted with DCM (3×50 ml). The organic extracts were combined and dried over anhydrous Na_2_SO_4_. After removal of the solvent, the crude product was charged into two-neck flask. Acetic acid (50 mL) and concentrated HCl (0.5 mL) were added and the mixture was refluxed for 4 h. Then the mixture was poured into water, extracted with hexane. The resulted crude compound was purified by silica gel chromatography using a mixture of DCM: PE (1：19)as the eluent to give a light yellow solid (3.85 g, 68%). ^1^H NMR (600 MHz, CDCl_3_, δ): 7.23 (d, *J* = 12 Hz, 8H), 7.19 (d, *J* = 6 Hz, 2H), 7.08 (d, *J* = 12 Hz, 8H), 6.94 (d, *J* = 6 Hz, 2H), 2.58 (t, *J* = 6 Hz, 8H), 2.21 (s, 6H), 1.60 (m, 8H), 1.35 (m, 24H), 0.89 (t, *J* = 6 Hz, 12H).

**Synthesis of Compound 5.**

In a dry two-neck round-bottomed flask, compound 4 (3.5 g, 3.75 mmol) was dissolved in 1,2-dichloroethane (50 mL) then add 10 ml DMF and placed under nitrogen atmosphere. The solution was cooled to 0 °C and stirred while phosphorus oxychloride in 1,2-dichloroethane (2.0 mL) was added dropwise. The mixture was stirred for 1 h at 25 °C, and then stirred at 100 °C overnight. After the reaction, the mixture was cooled to room temperature and poured into ice water. 1 M NaOH solution was added and extracted with DCM. The resulted crude compound was purified by silica gel column using a mixture of DCM:PE (2:3) as the eluent to give an orange solid (2.96 g, 80%). ^1^H NMR (600 MHz, CDCl_3_, δ): 9.76 (s, 2H), 9.59 (s, 2H),7.21 (d, *J* = 6 Hz, 8H), 7.10 (d, *J* = 12 Hz, 8H), 2.59 (t, *J* = 6 Hz, 8H), 2.26 (s, 6H), 1.61 (m, 8H), 1.31 (m, 24H), 0.88 (t, *J* = 6 Hz, 12H).

**Synthesis of Compound ID-MeIC.**

1,1-dicyanamethylene-3-indanone (490 mg, 2.53 mmol) was added into the mixture of compound 5 (1000 mg, 1.01 mmol) in anhydrous chloroform with pyridine (1 mL), the reaction was deoxygenated with nitrogen for 20 min and then refluxed for 10 h. After cooling to room temperature, the solution was poured into methanol and the precipitate was filtered off. Then it was extracted with DCM and washed with water. The crude product was purified by silica gel column using a mixture of DCM:PE (2:3) as the eluent to give a purple solid (1.10 g, 80%).^1^H NMR (600 MHz, CDCl_3_, δ): 8.80 (s, 2H), 8.67 (d, *J* = 6 Hz, 2H), 7.88 (d, *J* = 6 Hz, 2H), 7.73 (m, 4H), 7.26 (s, 2H), 7.23 (d, *J* = 6 Hz, 8H), 7.13 (2, *J* = 6 Hz, 8H), 2.59 (t, *J* = 6 Hz, 8H), 2.39 (s, 6H), 1.61 (m, 8H), 1.33 (m, 24H), 0.88 (t, *J* = 6 Hz, 12H).

**General measurement:**

UV-vis spectra were measured using a Perkin-Elmer Lambda-9 spectrophotometer. The ^1^H NMR spectra were collected on a Bruker AV400 and 600 spectrometers in deuterated chloroform solution with TMS as reference. Matrix-assisted time of flight mass spectrometry (MALDI-TOF) were performed on a Bruker Autoflex II / Compass 1.0 from Department of Materials Science and Engineering in Soochow University. Thermogravimetric analysis (TGA) was performed on a TA SDT 2960 instrument at a heating rate of 10 ^o^C/min from room temperature to 800 ^o^C under nitrogen. Cyclic voltammetry of polymer film was conducted in acetonitrile with 0.1 M of tetrabutylammonium hexafluorophosphate using a scan rate of 100 mV S^-1^. ITO, Ag/AgCl and Pt mesh were used as working electrode, reference electrode and counter electrode, respectively. Atomic force microscopy (AFM) images of the interfacial films were obtained using a Veeco Multimode V instrument. Grazing-incident wide-angle X-ray scattering (GIWAXs) were carried out at the SAXs/WAXs beamline of the Australian Synchrotron. The samples were prepared on ITO substrates in the same way as device fabrication with PEDOT : PSS as buffer layer. Samples were analyzed with an X-ray energy of 11 keV and a range of incident angles from Ω = 0.02 – 0.35 in 0.005 increments to allow signal optimization near the critical angle of the polymer film but below the critical angle of the substrate. And the detector location and the beam center were calibrated with a standard AgBr reference. Data from GIWAXs experiments were analyzed using a customized version of NIKA 2D based in IgorPro.


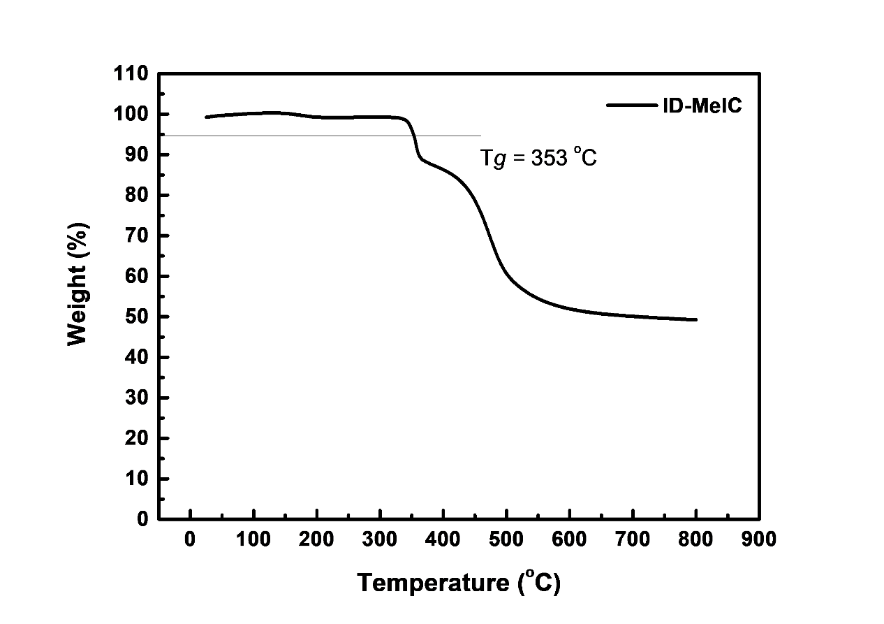


**Figure S1.** Thermogravimetric analysis (TGA) curve of ID-MeIC materials.


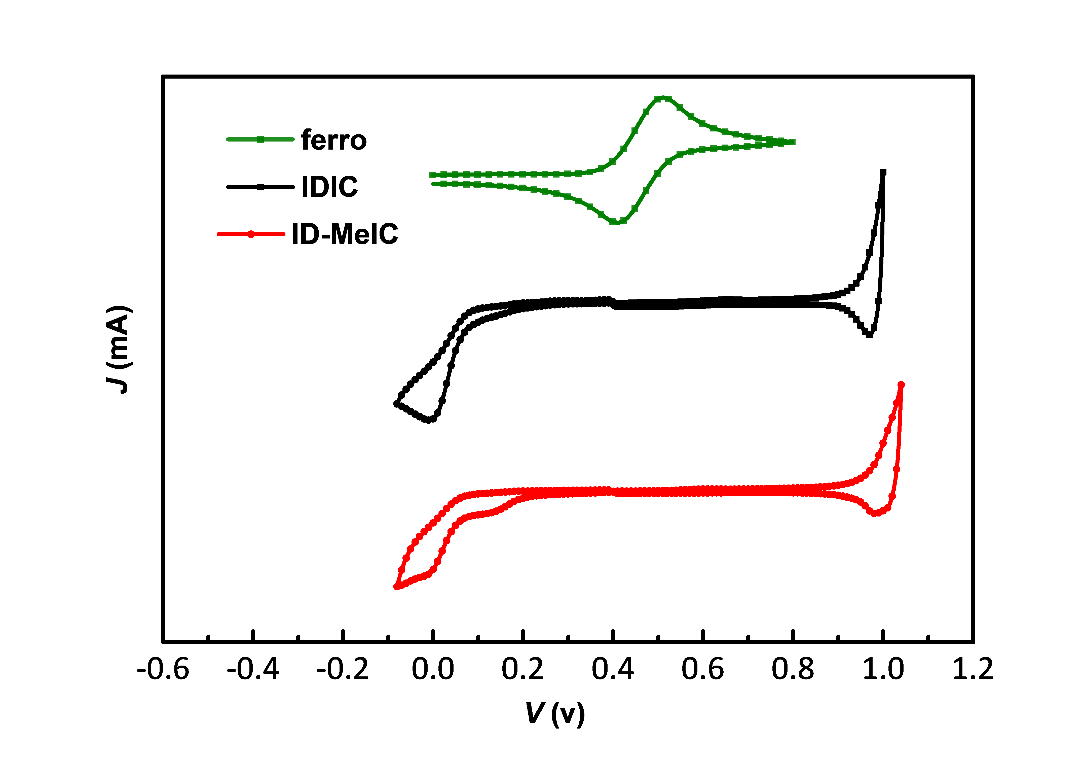


**Figure S2.** Cyclic voltammetry (CV) curve of IDIC and ID-MeIC film in diluted CH_3_CN solution with a scan rate of 100 mV s^-1^.


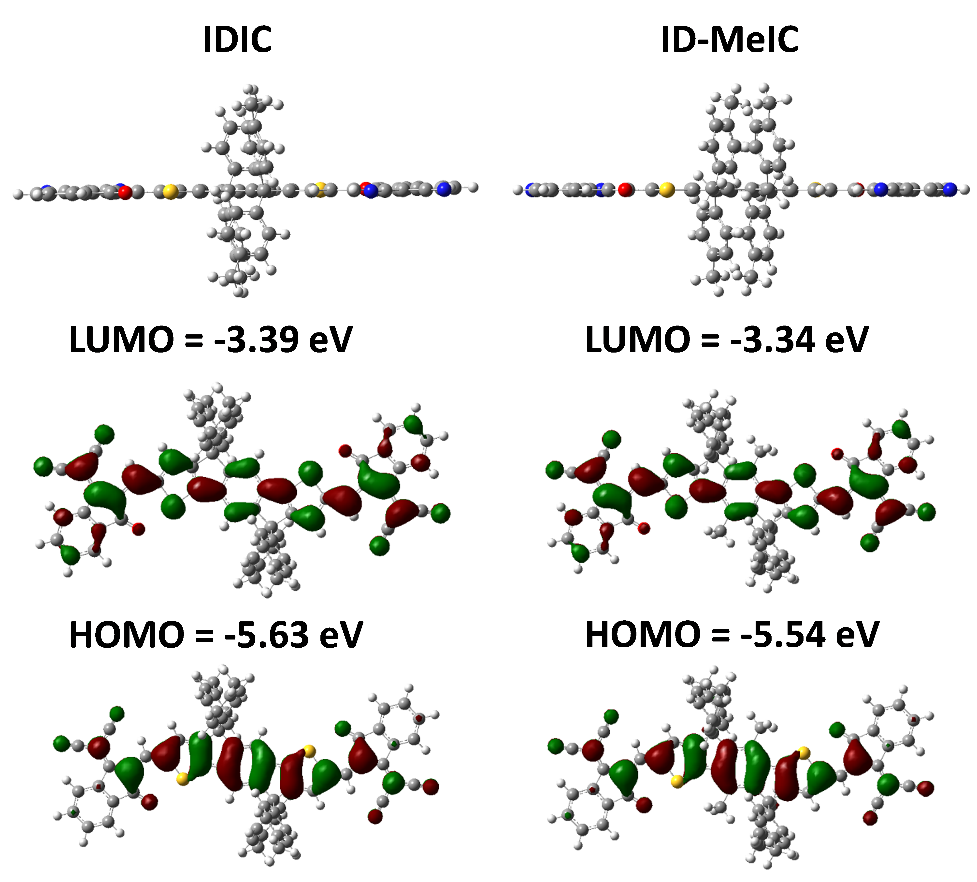


**Figure S3** Molecular geometries and the highest occupied molecular orbital and the lowest unoccupied molecular orbital (HOMO/LUMO) wave functions of the IDIC and

ID-MeIC.


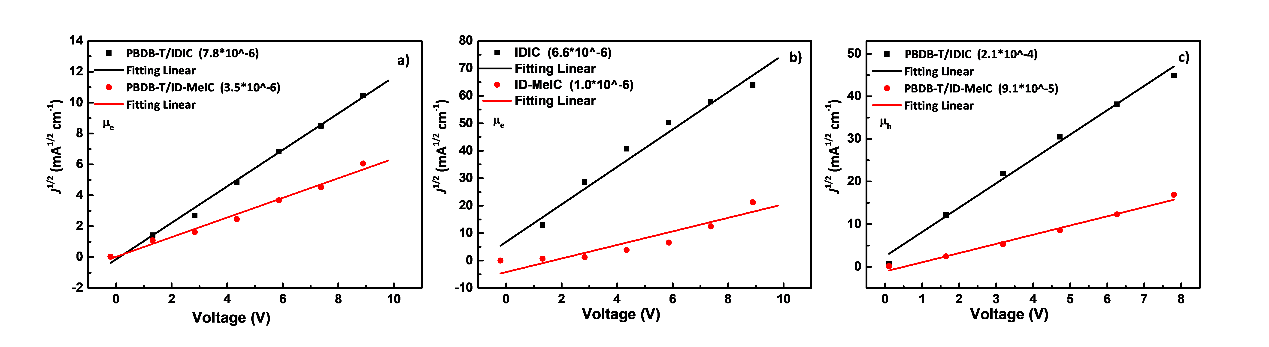


**Figure S4.** The IDIC and ID-MeIC pure film a) electron mobility; PBDB-T : IDIC and PBDB-T: ID-MeIC blends film curves b) electron mobility; c) hole mobility.

| Active layer | μ_h_ (cm^2^ V^-1^S^-1^) | μ_e_ (cm^2^ V^-1^S^-1^) | μ_h_/μ_e_ |
| --- | --- | --- | --- |
| PBDB-T/IDIC | 2.1×10^-4^ | 7.8×10^-6^ | 27 |
| PBDB-T/ID-MeIC | 9.1×10^-5^ | 3.4×10^-6^ | 26 |

| **Table S1.** The devices based on PBDB-T : IDIC active layer were fabricated with different D:A ratios | | | | | |
| --- | --- | --- | --- | --- | --- |
| Active Layer | D: A | *V*_OC_  (V) | *J*_SC_  (mA cm^-2^) | *FF*  (%) | PCE_max_/PCE^a^_avr_  (%) |
| PBDB-T : IDIC | 1:1 | 0.89±0.00（0.89） | 9.18±0.22（9.39） | 53±3  （56） | 4.73 / 4.34 |
|  | 0.8:1 | 0.89±0.00（0.89） | 8.65±0.47（8.82） | 52±2  （52） | 4.14 / 4.04 |
|  | 1:0.8 | 0.89±0.00（0.89） | 8.67±0.43（9.10） | 60±1  （61） | 4.94 / 4.67 |
|  | 2:1 | 0.88±0.01（0.89） | 7.33±0.48（7.50） | 45±3（48） | 3.25 / 3.01 |

| \| \| **Table S2.** The devices based on PBDB-T: ID-MeIC active layer were fabricated with different D:A ratios \| \| \| \| \| \| \| --- \| --- \| --- \| --- \| --- \| --- \| \| Active Layer \| D: A \| *V*_OC_  (V) \| *J*_SC_  (mA cm-2) \| FF  (%) \| PCE_max_/PCE^a^_avr_  (%) \| \| PBDB-T:ID-MeIC \| 1:1 \| 0.86±0.01  （0.87） \| 5.25±0.36（5.61） \| 55±2  （57） \| 2.82 / 2.52 \| \| 1:0.5 \| 0.87±0.01  （0.87） \| 5.30±0.60  （5.90） \| 58±2  （59） \| 3.04 / 2.72 \| \| 1:0.8 \| 0.87±0.01  （0.88） \| 5.63±0.64  （5.91） \| 59±2  （62） \| 3.22 / 2.90 \| \| 0.8:1 \| 0.86±0.02  （0.88） \| 5.74±0.83  （6.57） \| 54±1  （55） \| 3.17 / 2.68 \|   **Table S3.** The devices based on PBDB-T : ID-MeIC active layer were fabricated with different concentration at the D:A ratios for 1:0.8 \| \| \| \| \| \| \| --- \| --- \| --- \| --- \| --- \| --- \| --- \| --- \| --- \| --- \| --- \| --- \| --- \| --- \| --- \| --- \| --- \| --- \| --- \| --- \| --- \| --- \| --- \| --- \| --- \| --- \| --- \| --- \| --- \| --- \| --- \| --- \| --- \| --- \| --- \| --- \| --- \| --- \| --- \| \| Active Layer \| Concentration  (mg/mL) \| *V*_OC_  (V) \| *J*_SC_  (mA cm^-2^) \| *FF*  (%) \| PCE_max_/PCE^a^_avr_  (%) \| \| PBDB-T:ID-MeIC \| 10 \| 0.89±0.00（0.89） \| 10.10±0.59（10.69） \| 54±1  （55） \| 5.28 / 4.93 \| \| 15 \| 0.89±0.00（0.89） \| 13.01±0.31  （13.31） \| 43±3  （46） \| 5.49 / 5.09 \| \| 20 \| 0.89±0.00（0.89） \| 13.53±0.60（14.13） \| 38±3  （40） \| 5.11 / 4.74 \|   **Table S4.** The devices based on PBDB-T : ID-Me IC active layer were fabricated with different annealing temperature at the D:A ratios for 1:0.8 and the concentration for 15 mg/mL | | | | | | |
| --- | --- | --- | --- | --- | --- | --- | --- | --- | --- | --- | --- | --- | --- | --- | --- | --- | --- | --- | --- | --- | --- | --- | --- | --- | --- | --- | --- | --- | --- | --- | --- | --- | --- | --- | --- | --- | --- | --- | --- | --- | --- | --- | --- | --- | --- | --- | --- | --- | --- | --- | --- | --- | --- | --- | --- | --- | --- | --- | --- | --- | --- | --- | --- | --- | --- | --- | --- |
| Active Layer | Temperature  (^o^C) | DIO | *V*_oc_  (V) | *J*_sc_  (mA cm^-2^) | FF  (%) | PCE_max_/PCE^a^_avr_  (%) |
| PBDB-T:ID-MeIC | 70 | 0.5% | 0.90±0.00（0.90） | 13.67±0.46（14.13） | 50±1  （50） | 6.46 / 6.28 |
|  | 90 | 0.5% | 0.90±0.00（0.90） | 14.23±0.61（14.84） | 45±1  （46） | 6.15 / 5.90 |
